# The Acute Hepatic NF-κB-Mediated Proinflammatory Response to Endotoxemia Is Attenuated in Intrauterine Growth-Restricted Newborn Mice

**DOI:** 10.3389/fimmu.2021.706774

**Published:** 2021-09-01

**Authors:** Miguel A. Zarate, Robyn K. De Dios, Durganili Balasubramaniyan, Lijun Zheng, Laura G. Sherlock, Paul J. Rozance, Clyde J. Wright

**Affiliations:** Section of Neonatology, Department of Pediatrics, Children’s Hospital Colorado, University of Colorado School of Medicine, Aurora, CO, United States

**Keywords:** liver, innate immunity, LPS, NF-κB, cytokines, sepsis, intrauterine growth restriction

## Abstract

Intrauterine growth restriction (IUGR) is a relevant predictor for higher rates of neonatal sepsis worldwide and is associated with an impaired neonatal immunity and lower immune cell counts. During the perinatal period, the liver is a key immunological organ responsible for the nuclear factor kappa B (NF-κB)-mediated innate immune response to inflammatory stimuli, but whether this role is affected by IUGR is unknown. Herein, we hypothesized that the newborn liver adapts to calorie-restriction IUGR by inducing changes in the NF-κB signaling transcriptome, leading to an attenuated acute proinflammatory response to intraperitoneal lipopolysaccharide (LPS). We first assessed the hepatic gene expression of key NF-κB factors in the IUGR and normally grown (NG) newborn mice. Real-time quantitative PCR (RT-qPCR) analysis revealed an upregulation of both IκB proteins genes (*Nfkbia* and *Nfkbib*) and the NF-κB subunit *Nfkb1* in IUGR *vs.* NG. We next measured the LPS-induced hepatic expression of acute proinflammatory genes (*Ccl3*, *Cxcl1*, *Il1b*, *Il6*, and *Tnf*) and observed that the IUGR liver produced an attenuated acute proinflammatory cytokine gene response (*Il1b and Tnf*) to LPS in IUGR *vs.* unexposed (CTR). Consistent with these results, LPS-exposed hepatic tumor necrosis factor alpha (TNF-α) protein concentrations were lower in IUGR *vs.* LPS-exposed NG and did not differ from IUGR CTR. Sex differences at the transcriptome level were observed in the IUGR male *vs.* female. Our results demonstrate that IUGR induces key modifications in the NF-κB transcriptomic machinery in the newborn that compromised the acute proinflammatory cytokine gene and protein response to LPS. Our results bring novel insights in understanding how the IUGR newborn is immunocompromised due to fundamental changes in NF-κB key factors.

## Introduction

Worldwide, intrauterine growth restriction (IUGR) affects approximately 10% of all newborns (1). Compared to normally grown controls, growth-restricted newborns are at increased risk of infection early in life (2–5). IUGR has been associated with an impaired cellular and humoral immunity characterized by lower white blood cell counts (5–7) and proliferation rates (8, 9). Nevertheless, the molecular mechanisms by how IUGR increases susceptibility to infection in the offspring in the perinatal period are yet to be understood.

The transcription factor NF-κB is intrinsic among all living species (10–14) and coordinates a proinflammatory transcriptional program in response to various infectious stimuli. During the perinatal period, nuclear factor kappa B (NF-κB) signaling has a protective key role as a regulator of the acute phase innate immune response and immune organ development (15). Reduced NF-κB-mediated proinflammatory signaling has been associated with poor immune cell differentiation and activation (16), apoptosis (17), and decreased ability to respond to infection (18). Importantly, recent studies have linked the NF-κB innate immune transcriptome to multiple factors that control nutrient metabolism, cell survival, and organ development (19–21). These same pathways are severely affected in target organs in the growth-restricted fetus and neonate, including the liver (22–25).

Besides its metabolic functions during the fetal and perinatal period, the liver plays a key role in regulating the innate immune response due to its essential activity in clearing different antigens from the systemic circulation (26–29). NF-κB signaling in the liver is very active during development (30) due to its essential roles in tissue integrity (31), maturation of resident macrophages (12, 32) and lymphocytes (16, 29), and regulating the expression of key factors that are involved in hematopoiesis (33). Hepatic immune cells also contribute to the hepatic NF-κB-mediated innate immune response to different inflammatory stimuli (29, 34, 35). Our group has previously reported that the liver uniquely contributes to the innate immune response to an inflammatory challenge through the activation of the NF-κB signaling, which leads to the upregulation of proinflammatory cytokines across different developmental ages (36, 37). Furthermore, we have also shown that LPS-exposed fetuses can produce a NF-κB-dependent acute innate immune response, which occurs first and, to a greater degree, in the liver compared to other organs such as the lung and the skin (13). However, competent immune system development and activity requires an appropriate nutrient environment (38), particularly the NF-κB signaling in the liver, which is energy expensive (30). Therefore, an appropriate *in utero* environment is necessary for a correct NF-κB activity in the developing liver. Although the negative impact of IUGR has been described on liver metabolism and structure, its direct effects on the fetal or neonatal NF-κB-mediated hepatic innate immune system have never been explored.

With this in mind, a better understanding of the mechanisms that regulate acute hepatic NF-κB activation in the IUGR neonate could provide potential therapeutical approaches to prevent morbidity and mortality associated with *in utero* growth restriction. In the present work, we hypothesized that the newborn liver responds to IUGR by inducing fundamental changes in the expression of key members of the NF-κB signaling cascade, which leads to an attenuated acute innate immune response to an inflammatory challenge in a murine model of endotoxemia.

## Materials and Methods

### Ethical Approval

All animal experiments and procedures were approved by the University of Colorado Institutional Animal Care and Use Committee (00457) and conducted in compliance with the American Association for Accreditation for Laboratory Animal Care at the Perinatal Research Center at the University of Colorado School of Medicine (Aurora, CO, USA).

### Murine Model of Intrauterine Growth Restriction

Our IUGR model was based on a calorie-restricted diet and previously described by Chen and collaborators (39). This model has provided relevant insights on how the fetus adapts to undernutrition conditions *in utero*, and it has been extensively published (39–42). Briefly, 9–14-week-old C57BL/6 female mice (Jackson Laboratories, Bar Harbor, ME, USA) were allowed to mate with their male counterparts for 24 h (gestation day E0). Pregnant dams had *ad libitum* access to food from gestation days E1–E8. At the beginning of gestation day E9–18.5 (fetal tissue collection) or delivery (neonatal experiments), pregnant dams received a 50% calorie restriction diet (IUGR) or continued with an *ad libitum* (Control) feeding schedule (n = 5–6/group). At the end of the study (gestational day E18.5 or delivery), dams were euthanized with an IP overdose of sodium pentobarbital, and the fetal liver was collected, snap frozen in liquid nitrogen, and stored at −80°C. Fetal assessments were conducted in males and females, separately. Newborn mice (P0) were subsequently used for endotoxemia studies.

### Murine Model of Endotoxemia

Neonatal (P0) IUGR and normally grown (NG) C57BL/6 mice (n = 5–7/group) were exposed to a sublethal dose of intraperitoneal (IP) LPS (5 mg/kg; L2630, Sigma-Aldrich, St. Louis, MO, USA; 1 h), as previously reported by our group (36, 37, 43, 44). Similar number of animals from each litter were included as the unexposed (CTR) group. Animals were euthanized with an IP overdose of sodium pentobarbital, and liver regions were collected as described previously, snap frozen in liquid nitrogen, and stored at −80°C. Neonatal experiments were performed in male and female mice separately.

### mRNA Extraction and Quantitative Real-Time PCR

Neonatal (P0) hepatic messenger RNA (mRNA) from the right medial lobe was collected using the RNeasy Mini Kit (Qiagen, Valencia, CA, USA) and measured for purity and concentration using the NanoDrop (Thermo Fisher Scientific, Waltham, MA). mRNA was converted into complementary DNA (cDNA) using the Verso cDNA synthesis Kit (Thermo Scientific, Waltham, MA, USA). Relative mRNA levels were evaluated by quantitative real-time PCR using the TaqMan gene expression system (Applied Biosystems, Foster City, CA, USA). Hepatic gene expressions of the NF-κB machinery, namely, *Chuk* (Mm00432529_m1), *Ikbkb* (mm01222247_m1), *Nfkbia* (Mm00477798_m1), *Nfkbib* (Mm00456849_m1), *Nfkb1* (Mm00476361_m1), *Rela* (Mm00501346_m1), and *Rel* (Mm00485657_m1), and proinflammatory cytokines, namely, *Ccl3* (Mm99999057_m1), *Cxcl1* (Mm04207460_m1), *Il1b* (Mm01336189_m1), *Il6* (Mm00446190_m1), and *Tnf* (Mm00443258_m1) in the P0 neonate were assessed with predesigned exon-spanning primers using the StepOnePlus Real-Time PCR System (Applied Biosystems, Foster City, CA, USA). We used the murine housekeeping gene *18s* to normalize real-time quantitative PCR (RT-qPCR) results, and quantification was performed using the cycle threshold (ΔΔCt) method as described previously. Samples were run as duplicates, and data are expressed as fold change relative to the mean in the NG and CTR group, respectively.

### Detection of Hepatic TNF-α Protein Levels by ELISA

Liver tissues from IUGR and NG newborn (P0) exposed to IP LPS with their respective controls (CTR) were homogenized using the Bullet Blender (NextAdvance, Troy, NY, USA), and protein concentrations were measured using the bicinchoninic acid (BCA) assay. Hepatic mouse TNF-α values were determined by the mouse TNF alpha uncoated ELISA kit (88-7324, Invitrogen, Carlsbad, CA, USA) according to the manufacturers’ protocols. We included negative and positive controls to correct and validate this assay, respectively.

### Statistical Analysis

Statistical analysis was conducted with GraphPad Prism 8 software (GraphPad, San Diego, CA, USA). Calorie-restriction pregnant mice weight was analyzed as a one-way ANOVA (IUGR as a main factor) with time as repeated measurements, and differences between groups were determined by the Dunnett *post-hoc* test. IUGR fetal and neonatal parameters (number of fetuses and weight) were analyzed by Student’s *t-*test, whereas RT-qPCR data were analyzed by the Mann–Whitney *U* nonparametric test, since gene expression values did not follow the normal distribution. TNF-α ELISA data were analyzed by two-way ANOVA (IUGR and LPS as factors), and difference between groups were determined by Fisher’s least significant difference (LSD) test. Both sexes were analyzed separately. Statistical significance was declared at *p* < 0.05.

## Results

### Calorie Restriction Diet During Pregnancy Induces IUGR in Mice

**Figure 1:**
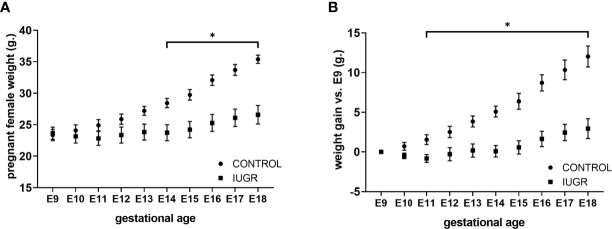
Calorie restriction reduces female weight maternal average gain weight during pregnancy. Pregnant C57BL/6 mice (n=6 per group) exposed to 50% calorie restriction diet (IUGR) or *ad-libitum* (CONTROL) throughout gestation were monitored daily for **(A)** weight, and **(B)** weight gain from E9 to E18.5. *P<0.05 *vs.* IUGR by l-way ANOVA with time as repeated measurements, and the Dunnet *Post-hoc* test. Values are represented as means ± SEM.

**Figure 2:**
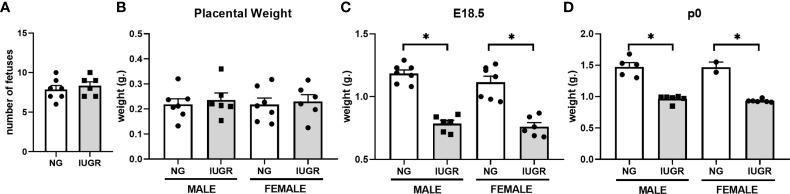
Intra-uterine growth restriction (IUGR) decreases fetal and neonatal weight. Fetal male and female **(A)** number, and average **(B)** placental weight, and **(C)** body weight were measured at E18.5. Each dot represents the mean per litter. **(D)** Neonatal male and female body weights were measured at P0. *P<0.05 *vs.* IUGR by Student’s *t* test. Values are represented as means ± SEM.

Calorie restriction-induced IUGR has been described in different animal models with various outcomes according to the pregnancy states. For this work, we used a modified IUGR murine protocol from Chen and collaborators (39). To validate our IUGR model, we first measured maternal weight and average weight gain across pregnancy starting at gestation age E9 (**Figures 1A, B**). Calorie restriction significantly reduced maternal weight from gestation age E14 (*p* < 0.01) and maternal average weight gain from gestation age E11 (*p* < 0.01) compared to *ad libitum* diet (Control) until the end of gestation. We further analyzed fetal and neonatal litter size and weight parameters (**Figures 2A** **–D**). Calorie-restriction IUGR did not induce any differences in fetal litter size (**Figure 2A**) or placental weight (**Figure 2B**). However, average litter weight at E18.5 was significantly reduced in both IUGR male and female fetuses compared to NG (*p* < 0.001). Similar differences continued in both IUGR male and female P0 neonatal mice compared to NG (*p* < 0.001). There were no sex differences in any parameters described previously.

### Calorie Restriction IUGR Modifies the Neonatal Hepatic NF-κB Transcriptomic Machinery

**Figure 3:**
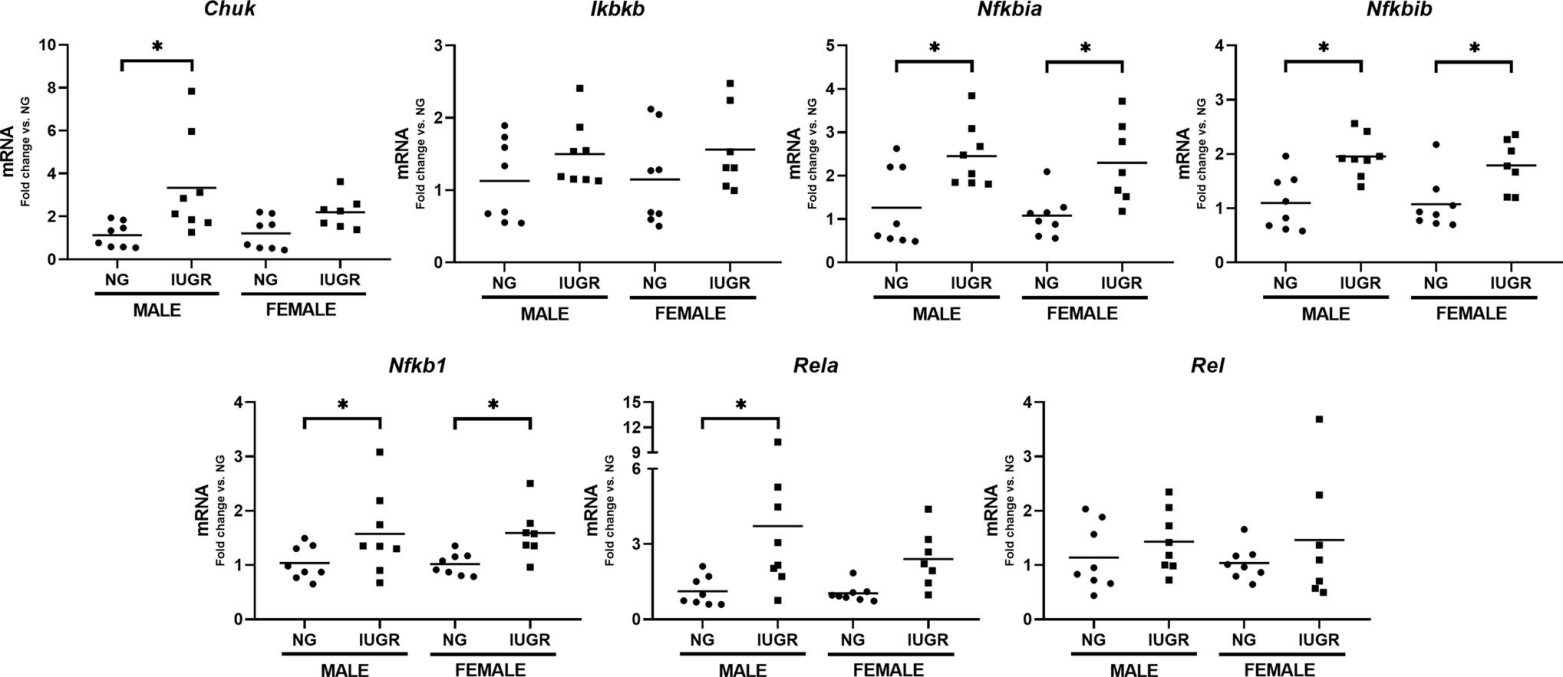
Intra-uterine growth restriction (IUGR) induces changes in the neonatal NF-κB transcriptomic machinery. IUGR and normally (NG) male and female (P0) lives were collected and measured for gene expression of NF-κB key factors. Mouse *18s* gene was used a housekeeping gene. *P<0.05 *vs.* NG by the Mann-Whitney test. Values are represented as fold change compared to their respective control (NG).

The innate immune system regulates the fetal and neonatal defense response against pathogen- (PAMPs) or damage-associated molecular patterns (DAMPs) due to a limited antigen exposure *in-utero* (15). The transcription factor NF-κB plays an essential role in the innate immune response (45, 46), and we have previously reported its importance in the liver, a key immune organ during the perinatal period. Activation of NF-κB-dependent proinflammatory genes *via* toll-like receptor (TLR) signaling (47–49) is regulated at different levels. Upregulation of the TLR-mediated proinflammatory cascade depends on the activation of the IκB kinases IKKα and IKKβ and the degradation of the inhibitory proteins IκBα and IκBβ, which will further allow the nuclear translocation of the NF-κB subunits p50, p65, and c-Rel (50–52). Although IUGR has been linked to an impaired cellular immunity in humans (8, 53, 54) and animals (23, 55, 56), the effects on the hepatic NF-κB transcriptomic machinery have never been explored. **Figure 3** shows the hepatic gene expression of different NF-κB signaling molecules in male and female P0 NG and IUGR neonates. Gene expression of the IκB kinase *Chuk* (IKKα) was significantly higher in the male IUGR neonate compared to NG (*p* < 0.05). Likewise, IUGR showed a significant upregulation of both NF-κB inhibitory protein genes *Nfkbia* (IκBα) and *Nfkbib* (IκBβ) in male and female neonates *versus* NG (*p* < 0.05). Similar effects were observed on the IUGR hepatic gene expression of the NF-κB subunits *Nfkb1* in both male and female (*p* < 0.05) and *Rela* only in male (*p* < 0.05) neonates compared to NG.

### The Acute Hepatic Innate Immune Response to an Inflammatory Challenge Is Attenuated in the Neonatal IUGR Liver

**Figure 4:**
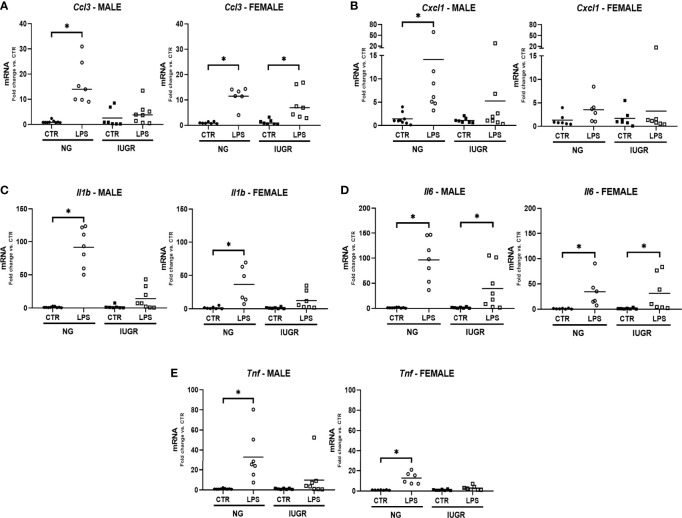
Intra-uterine growth restriction (IUGR) attenuates the hepatic gene expressions of pro-inflammatory cytokines in the LPS-exposed neonate. IUGR and normally grown (NG) male and female neonatal (P0) livers were collected and measured for hepatic gene expression of acute pro-inflammatory markers **(A)** *Ccl3*, **(B)** *Cxl1*, **(C)** *Il1b*, **(D)** *Il6*, and **(E)** *Tnf* at 1-hour post IP LPS. *P<0.05 *vs.* unexposed (CTR) by the Mann-Whitney test. Values are represented as fold change compared to their respective control (CTR).

We have previously reported that the liver uniquely contributes to the hepatic innate immune response when exposed to IP LPS through the expression of proinflammatory cytokines (36, 37). Since we detected significant changes in the hepatic IUGR NF-κB transcriptomic machinery, we decided to explore the effects of an IP LPS stimulation on the acute hepatic gene expression of key proinflammatory cytokines in the IUGR newborn. **Figure 4** shows the hepatic cytokine gene expression of *Ccl3*, *Cxcl1*, *Il1b*, *Il6*, and *Tnf* after 1 h IP LPS stimulation in the IUGR and NG male and female newborn. IP LPS exposure induced a significant upregulation of all hepatic proinflammatory cytokines in the NG male and female newborn compared to unexposed (CTR) (*p* < 0.05), with the exception of *Cxcl1* in the female mouse. Importantly, LPS exposure did not induce significant changes in the acute hepatic gene expression of *Il1b* and *Tnf* in both IUGR male and female mice and *Ccl3* and *Cxcl1* in male and female mice compared to IUGR CTR, respectively. There were no differences in the LPS-exposed hepatic *Il6* gene expression in the NG or IUGR neonate male and female mice compared to their respective CTR.

### Calorie Restriction IUGR Attenuates LPS-Induced Hepatic TNF-α Protein Expression Levels in the Newborn

**Figure 5:**
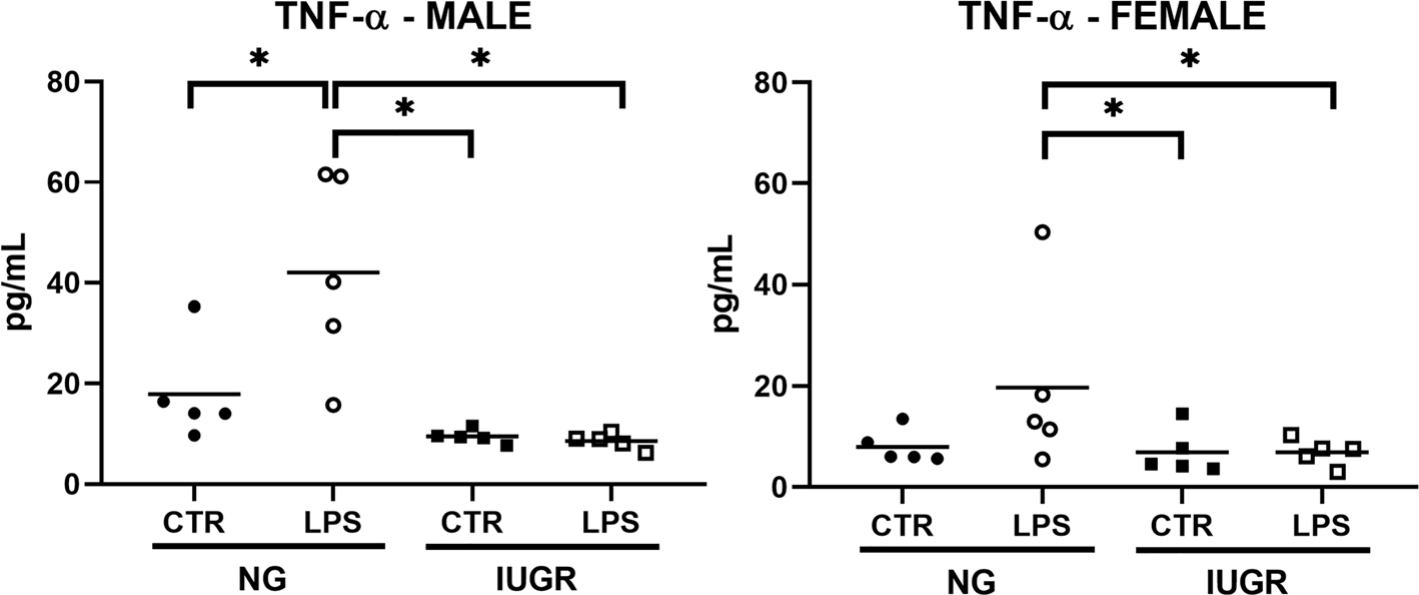
Intra-uterine growth restriction (IUGR) attenuates the hepatic TNF-α protein levels in the LPS-exposed newborn. IUGR and normally grown (NG) male and female neonatal (P0) livers were collected and measured for hepatic TNF-α (pg/mL) protein levels at 1-hour post IP LPS by ELISA. *P<0.05 by 2-way ANOVA (IUGR and LPS as factors), and Fisher’s LSD *Post-hoc* test. Values are represented as means.

Having observed important modifications in the NF-κB and acute proinflammatory cytokines transcriptome profile in the IUGR newborn compared to NG and the respective CTR after IP LPS exposure, we asked whether these transcriptional differences would affect the expression of at the protein level. Therefore, we decided to measure hepatic levels of TNF-α in the IUGR and NG neonate exposed to IP LPS by ELISA (**Figure 5**). Exposure to IP LPS significantly increased the hepatic TNF-α levels in the NG male neonate compared to NG CTR and to the LPS-exposed IUGR neonate (*p* < 0.01). TNF-α values in the IUGR male after LPS exposure did not produce any differences compared to the IUGR CTR. Similarly, LPS-exposed NG females tended to have higher hepatic TNF-α values compared to NG CTR (*p* = 0.066) and were significantly higher compared to both LPS-exposed and CTR IUGR newborn (*p* < 0.05).

## Discussion

This study reveals that calorie restriction IUGR is associated with transcriptional changes in the hepatic NF-κB signaling molecules and an attenuated acute proinflammatory innate immune response to IP LPS in the newborn. This work demonstrates three important findings (1): calorie restriction IUGR induces transcriptomic alterations in key members of the NF-κB signaling cascade in the neonatal liver. This is characterized by a significant upregulation of both IκB protein genes *Nfkbia* (IκBα) and *Nfkbib* (IκBβ), the NF-κB subunit *Nfkb1* in both male and female IUGR neonates, and the IκB kinase *Chuk* (IKKα) and the subunit *Rela* only in the IUGR male neonate. These fundamental changes were followed by (2) attenuated acute transcriptional proinflammatory cytokine gene response in the IUGR liver after LPS exposure (*Il1b* and *Tnf* in both male and female IUGR newborns and *Ccl3* and *Cxcl1* only in the male IUGR), and finally (3), a significant reduction in hepatic TNF-α values in both the LPS-exposed IUGR male and female compared to their respective LPS-exposed NG newborns.

The activation of the NF-κB signaling is mainly regulated at two levels (1): the NF-κB kinases (*Chuk* and *Ikbkb genes*), which phosphorylate the (2) IκB proteins (*Nfkbia* and *Nfkbib genes*) (57). The main role of these IκB proteins is to sequester the NF-κB subunits and prevent their nuclear translocation, thus inhibiting NF-κB-dependent gene transcription of proinflammatory cytokines. Our data indicate that calorie restriction IUGR induces upregulation of *Nfkbia* (IκBα) and *Nfkbib* (IκBβ) hepatic gene expression in male and female neonates and the IκB kinase *Chuk* (IKKα) in male mice. Effects on the NF-κB signaling due to alterations of the IκB proteins gene expression have been described previously. For instance, IκBα-protein-deficient mouse is reported to have a sustained NF-κB response with enhanced expression of proinflammatory cytokines (58), and newborns develop severe hematological disorders characterized by a surge in myeloid cells (59). Likewise, IκBβ-deficient cells show higher NF-κB activity after LPS exposure (60). On the other hand, gene upregulation of IκB proteins greatly inhibits the induction of the NF-κB signaling as reported by the use of immunosuppressants such as glucocorticoids (61) and nitric oxide (62). Similarly, overexpression of the IκB proteins significantly reduces NF-κB activity and proinflammatory cytokine (TNF-α and IL-1β) protein expression in myocardial cells (63) and impaired p65 nuclear translocation and reduced inflammation and necrosis in the liver (64).

Furthermore, we have found that the IUGR liver shows an upregulation of the NF-κB subunits *Nfkb1* in both male and female and *Rela* only in male neonates. The *Nfkb1* gene is reported to inhibit the expression of inflammatory genes such as *Il12b* (65), and *Cxcl9*, *Cxcl10*, and *Tnf* (66), and increase both gene and protein expression of the anti-inflammatory cytokine *Il10* (66) in macrophages. Likewise, p50 subunit (*Nfkb1*) homodimers can suppress the gene expression of NF-κB-dependent proinflammatory cytokines (67, 68). Upregulation of both hepatic IκB proteins (*Nfkbia* and *Nfkbib*) and *Nfkb1*genes in the IUGR newborns might fundamentally alter the acute proinflammatory innate immune response across development and contribute to the IUGR neonatal susceptibility to sepsis. The hepatic NF-κB transcriptome adaptations to IUGR presented here are novel and can bring important insights on the innate immune development and function in the newborn liver. Furthermore, the balance of the IκB kinases (IKKα and IKKβ) can also dictate the NF-κB transcriptome and activation (57), and their adaptations to IUGR also need to be further interrogated. In the present work, we have observed an upregulation of *Chuk* and *Rela* genes only in the IUGR male. The gene expression and activity of these key genes has been associated with the induction of the NF-κB signaling and upregulation of proinflammatory genes in response to an LPS challenge (69, 70). Although we detected upregulation of *Chuk* and *Rela* in IUGR male mice, which is consistent with a proinflammatory signaling, the expression of acute proinflammatory cytokines was not robust and, in some cases, not different compared to the unexposed mouse. Considering all this information, we speculate that the upregulation of *Chuk* and *Rela* occurs as a negative feedback mechanism to an impaired innate immune environment in the IUGR male liver.

Importantly, the transcriptomic changes previously described were associated with implications for the NF-κB-mediated acute proinflammatory cytokine gene response to LPS in IUGR neonatal mice. Our data indicate that IP LPS did not induce a robust acute hepatic proinflammatory cytokines gene expression in the IUGR newborn compared to the NG group. An attenuated acute proinflammatory transcriptomic response observed in the IUGR newborn mice was linked to a reduction in hepatic TNF-α cytokine values *versus* the LPS-exposed NG group.

Although neither IUGR male nor female newborns produced a strong acute hepatic proinflammatory innate immune response to IP LPS, this work reveals for the first time sex differences in this IUGR model. At the transcriptome level, there were more proinflammatory cytokines that did show any significant upregulation in the LPS-exposed IUGR newborn males compared to females (*Il1b*, *Tnf*, *Ccl3*, and *Cxcl1 vs .Il1b* and *Tnf*, respectively). Our data are supported by other groups who describe the male neonate as more susceptible to infections compared to females (71, 72), and this is possibly due to the actions of sexual hormones (73, 74). The mechanisms underlying the sex differences in the expression level of key members of the NF-κB signaling cascade in the IUGR newborn are yet to be determined, and this is a critical area for future work. At the protein level, however, acute hepatic TNF-α cytokine values did not differ from CTR after LPS exposure in both IUGR male and female. These data are novel and unique since this is the first time a group has measured the acute hepatic innate immune response to endotoxemia in the IUGR newborn at the transcript and protein level. Our results are supported by Fock et al., who described that peritoneal macrophage from protein-energy malnourished mice showed less NF-κB activation and TNF-α gene and protein expression after LPS challenge *in vitro* (18). On the other hand, IUGR is linked to an upregulation of proinflammatory cytokines in the neonatal brain (75–77), with even more exacerbated effects after LPS exposure (78), a complete different response observed in this study in the IUGR neonatal liver where the acute innate immune response to LPS did not differ from CTR. However, for the first time, our work shows how the IUGR liver might be compromised in its response to clearing systemic antigens, which can lead to detrimental effects on the overall health status of the newborn.

Our data are consistent with clinical data from groups who have measured different cytokine profiles in the IUGR human *versus* control (normally grown). For instance, Lindner et al. found that IUGR infants are immunocompromised due to lower concentrations of G-CSF and IL8 values (*Cxcl1* equivalent in the mouse) in the umbilical cord blood compared to normal-weight babies (79). Likewise, Neta et al. reported that small-for-gestational-age babies have lower interferon gamma (IFN-γ) and TNF-α levels in cord blood compared to normally grown babies (80), and Tröger et al. detected lower IL-6 and IL-10 protein levels in LPS-exposed IUGR blood cell cultures (81). Other reports indicate lower anti-inflammatory cytokine values (IL-10 and TGF-β) in the serum of IUGR infants *versus* their respective normally grown control (53). These studies along with our preclinical observations can provide relevant data to understand the neonatal IUGR innate immune response to a second hit stressor. However, other studies describe that IUGR babies might have a proinflammatory phenotype. For instance, Lausten-Thomsen et al. and Rocha et al. reported higher IL6 (82, 83), IL8 (83), TNF-α, and C-reactive protein (2) cytokine values in the IUGR umbilical cord blood compared to normally grown babies, respectively. Such inconsistencies might be attributed to the etiology of the IUGR phenotype (maternal, placental, or fetal origin).

Likewise, IUGR displays different cytokine responses according to the organ of study. For example, reports indicate a significant reduction in anti-inflammatory cytokine values in the IUGR blood and gastrointestinal tract (84) *versus* normal body weight neonates. On the other hand, IUGR skin (85), spleen (55), and placenta (86) display a greater proinflammatory cytokine expression in the fetus/neonate compared to their respective normally grown group. Given its relevant role as an immune organ in the perinatal period, we decided to investigate the hepatic NF-κB transcriptomic adaptations to calorie restricted IUGR. However, further work should focus on addressing the contribution of other organs to the NF-κB-dependent innate immunity in the IUGR newborn.

The exact mechanisms by which our calorie restriction IUGR model affects the hepatic expression of the IκB proteins and the *Nfkb1* genes and subsequent activation of proinflammatory responses are still unknown. We speculate that a calorie-restricted environment reduces the fetal ATP availability, a relevant substrate required by the IκB kinases for the activation of the NF-κB signaling. A reduction in ATP might lead to the upregulation of both IκB proteins and *Nfkb1* genes as a protective mechanism to starvation by the fetus at the expense of the activation of NF-κB-dependent proinflammatory cytokine responses and cellular enzymes, which are energy expensive. The inhibition of the NF-κB signaling will allow different fetal tissues to growth and differentiate, thus increasing chances for survival. As a consequence, these NF-κB transcriptomic modifications can severely compromise the acute hepatic innate response to an inflammatory challenge in the IUGR newborn that might be linked to susceptibility to sepsis and death.

The limitations of this study must be addressed. First, we only measured the IUGR acute hepatic proinflammatory innate immune response to LPS (1 h) and have not yet assessed these effects at later time points. Reports show that IUGR newborns and children can have prolonged immunity impairment (8, 9); thus, there is the need for longer LPS exposures in the IUGR neonate. However, for the first time, this work identified important differences in the IUGR hepatic NF-κB transcriptome and early proinflammatory cytokine responses to IP LPS. Furthermore, these fundamental changes could potentially be related to the multiple physiological outcomes observed in IUGR neonates such as increasing susceptibility to infections due to a blunted innate immunity. These findings will provide the foundations for testing the response to LPS at different range of exposures (doses), longer time points, and true models of sepsis. Second, we assessed the Toll-like receptor 4 (TLR4)-mediated hepatic innate immune response in the IUGR newborn. LPS exposure mimics bacterial infection, an event that is highly correlated with IUGR in the Neonatal Intensive Care Unit (87). Whether these effects will predict similar responses to other DAMPs or PAMPs are yet to be determined. This work provides relevant information that will be used for testing the response to other TLR agonists (e.g., CpGs, viral DNA, etc.) at different dosages and later time points. Finally, our assessments were done on whole liver tissues, and we did not account for specific-cell contributions on the innate immune response to LPS in the IUGR newborn. The main goal of this study involved the whole liver since IUGR might have effects on cellular number and/or density, which will make it difficult to compare to normally grown animals. Our results are relevant for future mechanistic studies (e.g., genetic, and pharmacological approaches) that will focus on understanding the role of hepatocytes and hepatic macrophages during TLR4-mediated proinflammatory episodes to create cell-specific therapeutical approaches in the IUGR newborn. Likewise, there are other factors involved in the response to endotoxemia. These factors include hypoxia (88), liver mass (89), nutritional status (90–92), and the presence of free radicals (93), among others. It is possible that these factors could contribute to the cytokine gene expression variability observed in the IUGR newborn exposed to IP LPS. To reduce variability, some IUGR studies identify the smallest or larger animals from the litter as IUGR and NG, respectively (94, 95). In this study, however, we used all newborns from IUGR and NG groups despite observing neonatal weight variability within the litter. Taking all these factors into consideration, it was expected to find some variability in the hepatic proinflammatory innate immune response to IP LPS. Still, significant differences between NG and IUGR were observed and reported in this study. Future studies will be conducted to elucidate the effects of all these factors in the IUGR newborn.

We conclude that the liver, a relevant innate immune organ during the perinatal period, responds to calorie restriction IUGR by inducing transcriptomic modifications on NF-κB key factors mainly by upregulation of the IκB proteins (*Nfkbia* and *Nfkbib*) and *Nfkb1* gene in both male and female newborns. These programming effects were followed by an attenuated hepatic TLR4-dependent acute innate immune cytokine response to IP LPS, which was also observed at the transcriptome and protein level (TNF-α) in IUGR newborns. Likewise, for the first time, we reported sex differences particularly on both NF-κB key factors (*Chuk* and *Rela*) and acute proinflammatory cytokine (*Ccl3* and *Cxcl1*) gene expression. Future work should center on understanding the relationship between sex hormones and the development of the innate immune system during IUGR. The present work shows how calorie restriction IUGR induces key modifications in the NF-κB transcriptomic machinery in the newborn mice and bring novel insights on how to treat neonatal sepsis by creating therapeutic approaches targeting specific NF-κB key factors.

## Data Availability Statement

The original contributions presented in the study are included in the article/supplementary material. Further inquiries can be directed to the corresponding author.

## Ethics Statement

The animal study was reviewed and approved by the University of Colorado Institutional Animal Care and Use Committee.

## Author Contributions

Conception and design of research: MZ, CW, PR, and LS. Data collection: MZ, CW, RD, DB, and LZ. Data analysis and interpretation: MZ, CW, PR, and LS. Elaboration of manuscript and figures: MZ and CW. All authors contributed to the article and approved the submitted version.

## Funding

The present work was funded by the National Institutes of Health grants R01-HL132941 (CJW), R01-HL132941-02S1 (MZ), R01-HD093701 (PR), DK088139 (PR), and T32HD007186 (MZ Trainee, PR PI).

## Conflict of Interest

The authors declare that the research was conducted in the absence of any commercial or financial relationships that could be construed as a potential conflict of interest.

The reviewer SM has declared a past collaboration with some of the authors RD, LS, PR, and CW to the handling editor at the time of review.

## Publisher’s Note

All claims expressed in this article are solely those of the authors and do not necessarily represent those of their affiliated organizations, or those of the publisher, the editors and the reviewers. Any product that may be evaluated in this article, or claim that may be made by its manufacturer, is not guaranteed or endorsed by the publisher.
